# Vaccine-induced immune thrombotic thrombocytopenia presenting as a mimic of heparin-induced thrombocytopenia in a hemodialysis patient receiving ChAdOx1 nCoV-19 vaccine

**DOI:** 10.1080/0886022X.2022.2098772

**Published:** 2022-07-12

**Authors:** Yi-Ling Lin, Chen-Yuan Lin, Jiung-Hsiun Liu

**Affiliations:** a School of Medicine, China Medical University, Taichung city, Taiwan; b Division of Hematology and Oncology, Department of Internal Medicine, China Medical University Hospital, Taichung City, Taiwan; c Division of Nephrology and Kidney Institute, Department of Internal Medicine, China Medical University Hospital, Taichung City, Taiwan

Dear editor,

Vaccines against severe acute respiratory syndrome coronavirus 2 (SARS-CoV-2) have become the most vital countermeasure against the coronavirus 2019 (COVID-19) pandemic. However, numerous complications have been reported, such as fever, headache, myalgia, and rash, after receiving an anti-COVID-19 vaccine [1]. One novel syndrome reported is that of ‘vaccine-induced immune thrombotic thrombocytopenia (VITT)’ after the injection of ChAdox1 nCoV-19 (AstraZeneca) or Johnson & Johnson adenoviral vaccines, mostly since March 2021 [2]. A few cases of major thrombosis with concurrent thrombocytopenia after vaccination of mRNA-1273 (Moderna) and Pfizer-BioNTech have also been documented by the UK regulatory agency, although diagnoses of VITT have not been confirmed. Cases of VITT after a second dose have also been reported, although at a much rarer rate than after first dose [1]. The proposed mechanism of VITT is the production of antibodies against platelet factor 4 (PF4), resulting in massive platelet aggregation [2]. This complication is critical since it may lead to life-threatening events.

Thrombocytopenia probably occurred in many causes in dialysis patients [3]. A special trigger of thrombocytopenia owing to heparin-induced thrombocytopenia (HIT) can occur in hemodialysis (HD) patients. Up to 12% of dialysis patients develop HIT due to continuous exposure to heparin [3]. HIT is the most frequent drug-induced type of thrombocytopenia and is induced by IgG antibodies recognizing epitopes on the positively charged chemokine PF4 within PF4-polyanion complexes. Patients who receive heparin for 7–10 days are at high risk of developing HIT [4].

As VITT and HIT are both mediated by platelet-activating antibodies against PF4 [2], VITT likely mimics HIT on the basis of both clinical and serological evidence. This is likely making for confusion between VITT and HIT in HD patients. Herein, we report on a rare VITT case in an 85-year-old male on HD who received the AstraZeneca (AZ) vaccine.

An 85-year-old male with a history of end-stage renal disease undergoing regular HD with low-molecular-weight heparin since 2016, presented to the emergency department with a headache 7 days after a second dose of the AZ vaccine (Siam Bioscience; Bangkok, Thailand; 10 doses per vial − 0.5 mL per dose). Tracing back his medical history, his serum platelet counts had been within normal limits (150,000/mm^3^ to 450,000/mm^3^) as indicated in routine blood tests before the second dose of the AZ vaccine. Notably, the patient did not suffer from any thrombocytopenia or thromboembolism but only mild fever and myalgia after his first dose of the AZ vaccine, which was administered 3 months prior to the second dose. In addition, there was no previous history of any autoimmune or thrombotic disease.

On physical examination, no neurologic deficits were evident and computed tomography of the brain was unremarkable (Figure 1). A complete blood count showed severe thrombocytopenia (platelet count, 2000/mm^3^) on the 7th day after vaccination. Given the unexplained thrombocytopenia, he was admitted to our hematology ward for further survey.

**Figure 1.**
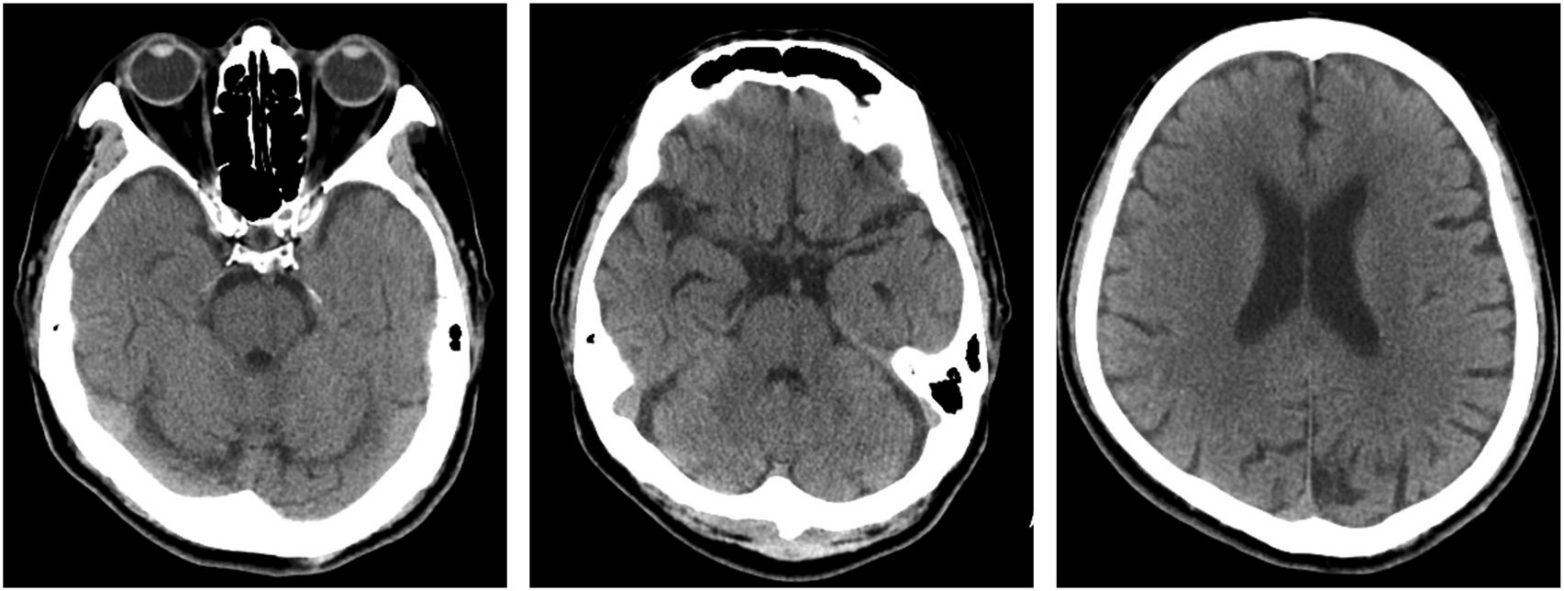
Non-enhanced computed tomography (CT) of the brain showed no established major vessel vascular territory infarct.

A review of his dialysis chart indicated that heparin was suspended due to the development of gastrointestinal bleeding, 3 weeks prior to his second vaccination dose. After the bleeding episode resolved, unfractionated heparin was administered 16 days later. As thrombocytopenia occurred 7 days after the second dose vaccination, non-heparin HD was performed after hospital admission. No obvious thrombotic event was recorded, apart from one time of dialysis circuits clotting in trace status after admission. A series of examinations were done (Figure 2). Initial hematological parameters upon admission showed thrombocytopenia with platelet count less than 10,000/mm^3^ and a D-dimer peak greater than 4.5 mg/L. Nevertheless, the prothrombin time (9.5 s), activated partial prothrombin time (26.1 s) and international normalized ratio (0.78) were within normal ranges. He was afebrile and leukocytosis was not found (white blood counts, 6200/mm^3^). Both Helicobacter pylori. and virus screening gave unremarkable results. Stable hemoglobin levels (10.1 g/dL), preserved ADAMTS 13 activity and no schistocytes found on his blood smear did not support atypical hemolytic uremic syndrome or thrombotic thrombocytopenia (TTP). Serum antinuclear antibody and antiphospholipid antibody were also examined, and evidence was insufficient to support the possibility of rheumatic disease or other immunologic disease. His medication history was reviewed and other drug-related thrombocytopenia was excluded. An abdominal ultrasound showed no evidence of liver cirrhosis and no splenomegaly. Given his symptoms, vaccination history, dialysis history and the laboratory findings, VITT or HIT was highly suspected. The autoantibody of PF4 was checked by enzyme-linked immunosorbent assay (ELISA) on his hospital admission day and found to be positive with an optical density (OD) value of 0.428 (positive as OD >0.4), confirming our hypothesis.

**Figure 2.**
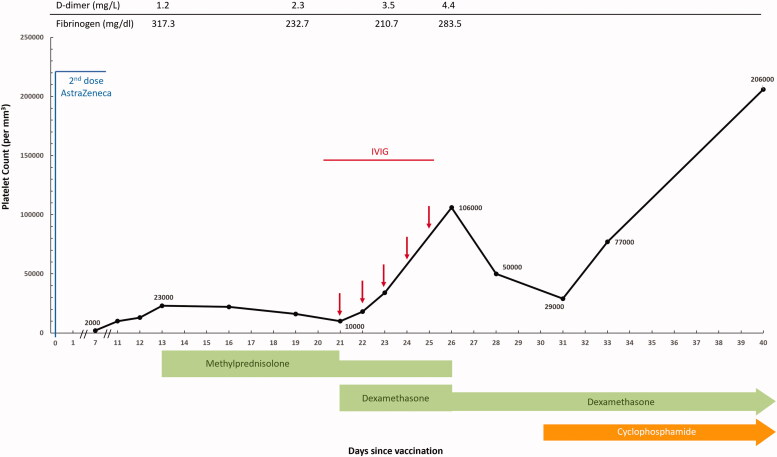
Clinical and laboratory data for the patient.

Initially, he was treated with intravenous (IV) methylprednisolone (1 mg/kg/day, 70 mg daily) for a week. Due to poor response, treatment of IV immune globulin (2 mg/kg/daily for 5 days) was initiated, and combined with dexamethasone (5 mg daily). Anti-PF4 antibody level was rechecked and showed an OD value of 0.702. Given that the platelet counts did not rise to an acceptable level despite previous treatment, oral cyclophosphamide (50 mg daily for 10 days) was prescribed as an immunosuppressant. IV dexamethasone was continued in tapering doses. The platelet count was normalized (206,000/mm^3^) within 4 weeks and no clinical evidence suggested that thrombosis was increasing. The pattern of laboratory parameters observed along with clinical regimens are shown in Figure 2. In addition, non-heparin HD was performed throughout his hospitalization because no circuit clotting issue occurred after platelet counts normalized. He was eventually discharged with good health status. No additional events or rehospitalization were observed during 2 months of outpatient follow-up thereafter.

The epidemiology of VITT is rapidly evolving, with several formulated guidelines for diagnosis and management under development. The case we present fulfilled four of the five diagnostic criteria from the UK Hematology Expert Groups (Supplementary Item S1, [5]), including the onset of symptoms: 5–30 days after vaccination against SARS-CoV-2, thrombocytopenia (platelet count <150,000/mm^3^), a D-dimer level greater than 4 mg/L and the presence of antibodies to PF4 detected, but the fifth criterion, thrombosis, was not documented. The case was classified as a highly probable VITT based on these criteria. Other possible causes of thrombocytopenia, including previous infection, thrombophilia, TTP, autoimmune disease and other medications were specifically excluded.

For thrombocytopenia in patients on HD, HIT must be a differential diagnosis. Dialysis patients are at risk of HIT 7–10 days after receiving heparin, with clinical presentations of acute thrombocytopenia and thrombotic events [3]. The widely used 4 T score can be helpful in the diagnosis of HIT; the score evaluates four indicators: the degree of thrombocytopenia, timing, thrombotic events, and alternative causes of HIT (Supplementary Item S2, [6]). Treatment of typical HIT requires cessation of heparin and an initiation of alternative anti-coagulants [4]. In our case, the total 4 T score was 3 points, indicating the possibility of HIT is low. Given our patient’s presenting history and thorough examinations, VITT was diagnosed and treated, with good outcome after standard regimens.

As shown in this case, a rapidly progressive thrombocytopenia developing on dialysis patients after the AZ vaccination may clinically mimic HIT. Our findings expand upon prior reports in which anti-PF4/heparin antibodies, referred to as heparin-induced antibodies (HIA), were shown to be associated with both VITT and HIT [4]. A few published studies have demonstrated that some individuals remain asymptomatic without thrombocytopenia or thromboses but have detectable HIA [7]. In addition, the prevalence of HIT in HD is less than 1% whereas that of HIA is higher [8]. Previous reports on the prevalence of HIA have been highly variable in HD patients [7]. Palomo et al. [7] have found that 17.9% of dialysis patients in their center developed HIA, while Matsuo et al. [9] reported that 2.3% of dialysis patients were positive for HIA at four kidney centers. A high prevalence of HIA in HD patients may obscure identification of VITT in HD if using only the indicator of HIA. For those under dialysis, the case definition criteria from the UK Hematology Expert Groups might be a more comprehensive approach to diagnosing VITT.

All current treatment recommendations for VITT are based on extrapolation from autoimmune thrombotic thrombocytopenia. The consensus of most experts is that a second-line therapy of immunomodulators be applied when first-line therapy (e.g. steroids) does not raise the platelet count to a safe level. Since our study indicated a beneficial effect of cyclophosphamide, we suggest it could be considered in severely-affected patients who do not respond to standard therapy. The optimum treatment schedule of cyclophosphamide remain to be determined.

It is challenging to prove definitive causality in the case we present since VITT is a novel syndrome and not yet fully understood. An additional survey of thrombosis or microthrombosis could further verify a definitive diagnosis for VITT.

VITT has emerged as a rare but devastating complication. The presented case highlights the need for timely recognition of VITT to prevent devastating complications in an HD patient receiving an anti-COVID-19 vaccination.

## Supplementary Material

Supplemental Material
