# Supplementary material for: Vaccine-induced immune thrombotic thrombocytopenia presenting as a mimic of heparin-induced thrombocytopenia in a hemodialysis patient receiving ChAdOx1 nCoV-19 vaccine
Source: Ren Fail. 2022 Jul 12;44(1):1130–3. doi: 10.1080/0886022X.2022.2098772 (PMC9291670; doi:10.1080/0886022X.2022.2098772)
Supplement: Supplemental Material [file IRNF_A_2098772_SM3093.pdf]

Item S1. Case definition criteria from the UK Hematology Expert Groups

**Case definition criteria**	• Onset of symptoms 5-30 days post COVID-19 vaccine (or up to 42 days if isolated DVT/PE)<br>• Presence of thrombosis<br>• Thrombocytopenia (platelet count <150,000/mm^3)<br>• D dimer >4 mg/L<br>• Positive anti-PF4 Abs ELISA assay
**Definite VITT**	• Meets all five criteria
**Probable VITT**	• D dimer >4 mg/L but one criterion not fulfilled (timing, thrombosis, thrombocytopenia, anti-PF4 Abs), or<br>• D dimer unknown or 2-4 mg/L with all other criteria present
**Possible VITT**	• D dimer unknown or 2-4 mg/L with one other criterion not fulfilled, or<br>• Two other criteria not fulfilled (timing, thrombosis, thrombocytopenia, anti-PF4 Abs)
**Unlikely**	• Platelet count <150,000/mm^3 without thrombosis with D dimer <2 mg/L, or<br>• Thrombosis with platelet count >150,000/mm^3 and D dimer <2 mg/L, regardless of anti-PF4 Ab result, and/or<br>• Alternative diagnosis more likely

*Adapted from Pavord et al. [5]

DVT deep vein thrombosis, *PE* pulmonary embolism, *PF4* platelet factor 4, *Abs* antibodies

Item S2. 4T Scoring System for Evaluating the Probability of HIT

Variable	Score		
	2	1	0
**Acute thrombocytopenia**	Platelet count decrease of >50% and nadir ≥20,000/mm^3	Platelet count decrease of 30–50% or nadir 10,000–19,000/mm^3	Platelet count decrease of <30% or nadir ≤10,000/mm^3
**Timing of onset**	Day 5–10, or day 1 if recent heparin exposure	>Day 10 or unclear exposure	≤Day 4 with no recent heparin exposure
**Thrombosis**	New thrombosis or anaphy-lactoid reaction after heparin bolus	Progressive or recurrent thrombosis	None
**Other cause of thrombocytopenia**	None	Possible	Definite
**Total score**	6–8, indicating high score	4 or 5, indicating intermediate score	0–3, indicating low score

* Adapted from Lo et al. [6]
